# Prediction of breast cancer using blood microbiome and identification of foods for breast cancer prevention

**DOI:** 10.1038/s41598-023-32227-x

**Published:** 2023-03-29

**Authors:** Jeongshin An, Jinho Yang, Hyungju Kwon, Woosung Lim, Yoon-Keun Kim, Byung-In Moon

**Affiliations:** 1 Institute of Convergence Medicine Research, Ewha Womans University Mokdong Hospital, Ewha Womans University School of Medicine, 1071 Anyangcheon-ro, Yangcheon-gu, Seoul, 07985 Republic of Korea; 2 Department of Surgery, Ewha Womans University Mokdong Hospital, Ewha Womans University School of Medicine, 1071 Anyangcheon-ro, Yangcheon-gu, Seoul, 07985 Republic of Korea; 3 MD Healthcare, Room 1303, Woori Technology Inc. building, Sangam-dong, World Cup Buk-ro 56-gil, Mapo-gu, Seoul, Republic of Korea; 4 Department of Occupational Health and Safety, Semyung University, 65 Semyung-ro, Jecheon, Chungcheongbuk-do 27136 Republic of Korea

**Keywords:** Cancer, Genetics, Microbiology

## Abstract

The incidence of breast cancer (BC) is increasing in South Korea, and diet is closely related to the high prevalence of BC. The microbiome directly reflects eating habits. In this study, a diagnostic algorithm was developed by analyzing the microbiome patterns of BC. Blood samples were collected from 96 patients with BC and 192 healthy controls. Bacterial extracellular vesicles (EVs) were collected from each blood sample, and next-generation sequencing (NGS) of bacterial EVs was performed. Microbiome analysis of patients with BC and healthy controls identified significantly higher bacterial abundances using EVs in each group and confirmed the receiver operating characteristic (ROC) curves. Using this algorithm, animal experiments were performed to determine which foods affect EV composition. Compared to BC and healthy controls, statistically significant bacterial EVs were selected from both groups, and a receiver operating characteristic (ROC) curve was drawn with a sensitivity of 96.4%, specificity of 100%, and accuracy of 99.6% based on the machine learning method. This algorithm is expected to be applicable to medical practice, such as in health checkup centers. In addition, the results obtained from animal experiments are expected to select and apply foods that have a positive effect on patients with BC.

## Introduction

Breast cancer (BC) is a severe health problem worldwide and is the most prevalent cancer among women^1^. In Korea, BC has become the most common cancer in women in 2020, and the proportion of patients with BC is increasing every year^2^. Despite effective treatment, BC is highly prevalent. Therefore, we aimed to focus on preventing BC occurrence by studying the causes of BC. Hereditary BC comprises only 5–10%^3^ of all BCs. Dietary issues are another cause of BC^4^. Dietary patterns are either directly or indirectly related to the microbiome and BC^5^ is associated with changes in eating habits^6^: higher consumption of red or processed meats and foods with a high glycemic index is associated with an increased risk of BC^4^. In this study, the influence of the microbiome, which is closely related to eating habits, was analyzed in Korean patients with BC.

Radiographic examination and histopathological biopsy of breast tissues are currently used to diagnose BC^7^. A biopsy is performed through imaging examination when the Breast Imaging-Reporting and Data System (BI-RADS) is category 4A or higher^8^, with the patients being diagnosed with BC if cancer cells are found. Although it is the most accurate way to identify cancer in tissues, the size of the cancer must be large enough to be detected by imaging. Furthermore, benign mimicry of malignant tumors presents difficulties in diagnosis, even for the most experienced pathologist^9^. Another option for diagnosis is the analysis of microbiome patterns in the blood, and a previous study has assigned unique patterns in the microbiome to each cancer^10^. In addition, the microbiome composition is altered by various factors such as diet, infection, and lifestyle reflecting individual environment^11^.

An ideal diagnostic program would detect high-risk groups of BC while increasing the possibility of early diagnosis of cancers that can be missed in imaging tests. We attempted to diagnose BC using a noninvasive microbiome ratio. This was verified using data from Korean patients with BC, and the accuracy was compared using the three verified methods which are stepwise, linear discriminant analysis (LDA) and LDA effect size (LEfSe), and machine learning algorithm. In particular, machine learning methods on data are advantageous for diagnosis because they are flexible and scalable and can be trained to detect complex nonlinear relationships between variables that cannot be easily captured by traditional statistical methods^12^. In addition, experiments using animals on prebiotics were showed which food is meaningful in breast cancer. These probiotics may be useful to prevent BC in patients. In this process, we analyzed the microbiome using bacterial extracellular vesicles (EVs) from the blood. Bacterial EVs are nanometer-sized organs that contain bioactive nucleic acids, lipids, and proteins inside a lipid bilayer^13^. Since EVs are found floating in the bloodstream, they are a great tool to analyze the microbiome in the body^14^.

We analyzed microbiome data from patients recruited by more than two institutions and developed a predictive model for BC diagnosis. This study compared microbiome patterns through three statistical methods, including machine learning, and focused on identifying prebiotics that is helpful for BC prevention by validating this model.

## Results

### Diversity

**Figure 1:**
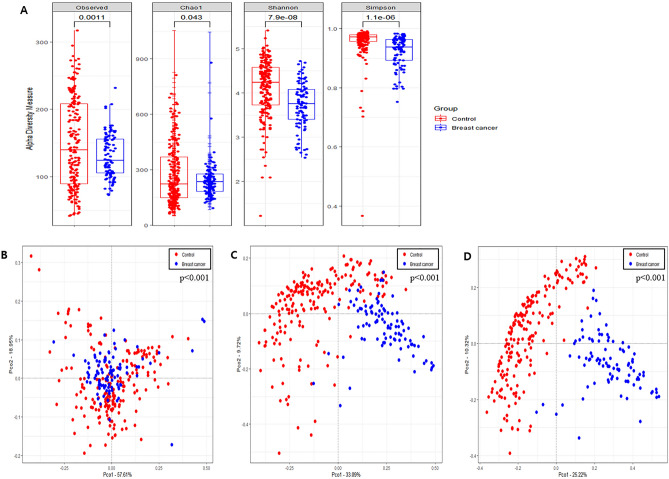
Difference of diversity between breast cancer and healthy control. (**A**) Alpha diversity including Observed OTUs, Chao1 index, Shannon index and Simpson index. Principal Coordinate Analysis (PCoA) at the (**B**) phylum level, (**C**) family level, and (**D**) genus level.

Alpha diversity was analyzed using the observed OTUs, Chao1 index, Simpson index, and Shannon index for richness and evenness. The observed OTUs, Simpson index, and Shannon index in HC were significantly higher than those in BC; however, the Chao1 index in HC was significantly lower than that in BC (p < 0.05) (Fig. 1A). Rarefaction curves based on Chao1 index for the sequences per sample are shown in Supplementary Fig. 1.

The PCoA plot based on the Bray–Curtis dissimilarity matrix showed significant differences in beta diversity between BC and HC groups by PERMANOVA at all levels (p < 0.001) and distinct clustering at lower taxonomic levels (genera and species) (Fig. 1B–D, Supplementary Fig. 2A–C).

### Metagenome profiles of serum samples

**Figure 2:**
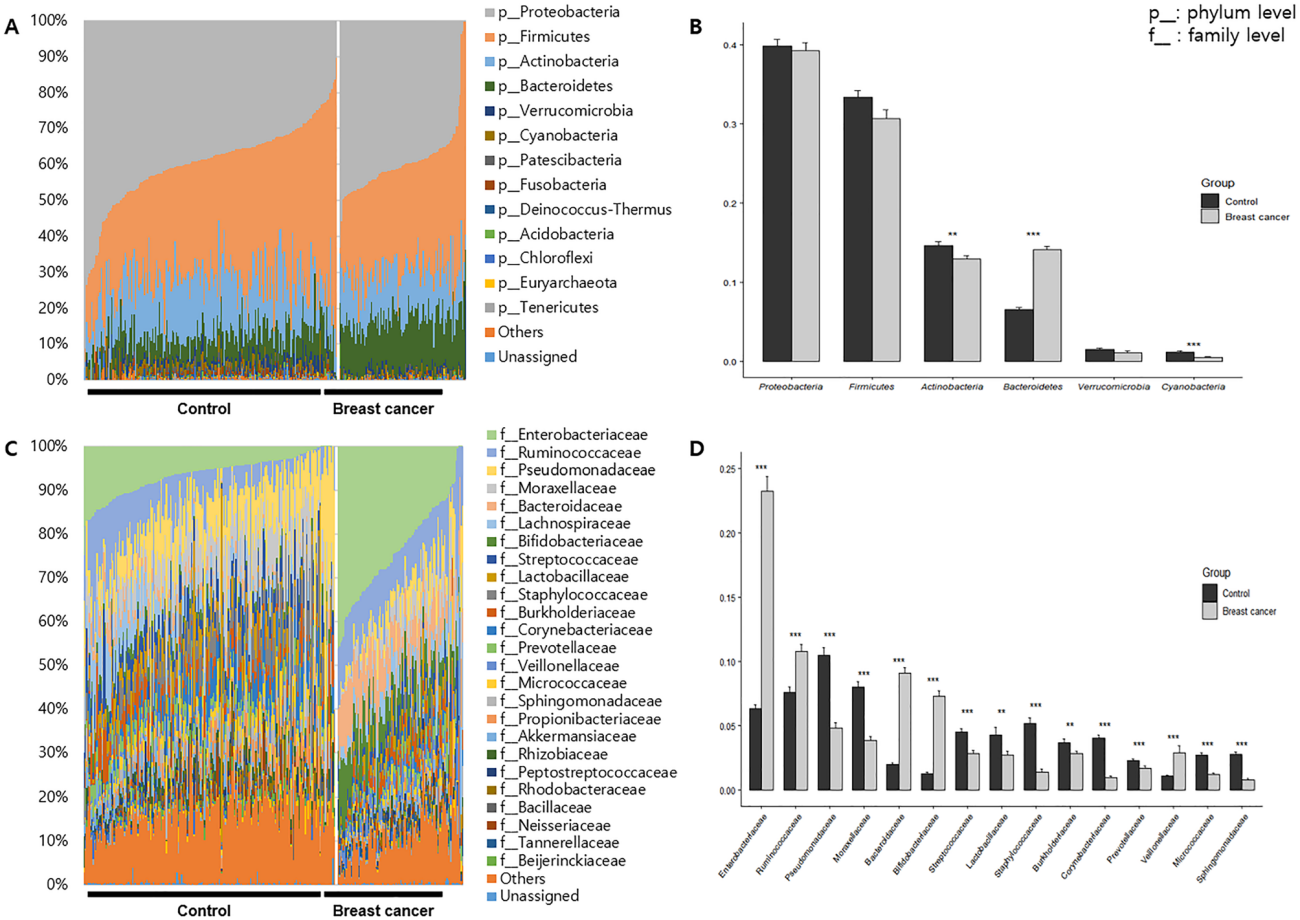
Metagenome profiles in serum of patients with breast cancer and healthy controls. (**A**) The composition of microbiome at the phylum level, (**B**) the difference of main phyla, (**C**) the composition of microbiome at the family level, and (**D**) significantly different taxa at the family level.

At the phylum level, Proteobacteria, Firmicutes, Actinobacteria, and Bacteroidetes were dominant in HC and BC groups, accounting for over 94% of the bacterial abundance (Fig. 2A). *Actinobacteria*, *Bacteroidetes*, and *Cyanobacteria* abundance differed significantly between HC and BC groups (*p* < 0.05): 14.6 ± 7.2%, 6.6 ± 3.7%, and 1.2 ± 1.6% in HC and 12.9 ± 4.1%, 14.2 ± 3.7%, and 0.5 ± 0.6% in BC for *Actinobacteria*, *Bacteroidetes*, and *Cyanobacteria*, respectively (Fig. 2B).

At the class level, the abundances of *Gammaproteobacteria*, *Actinobacteria*, and *Clostridia* were higher than 10% in both HC and BC groups (Supplementary Fig. 3A). *Gammaproteobacteria*, *Clostridia*, *Bacteroidia*, *Negativicutes*, and *Coriobacteriia* abundances were significantly lower in HC than in BC, whereas *Bacilli*, *Actinobacteria*, *Alphaproteobacteria*, and *Oxyphotobacteria* were significantly higher in HC than in BC (Supplementary Fig. 3B).

At the order level, 16 and 13 taxa accounted for > 1% in HC and BC, respectively. *Pseudomonadales* was dominant in HC with 18.5 ± 10.6% abundance and *Enterobacteriales* was dominant in BC with 23.3 ± 11.2% abundance (Supplementary Fig. 3C). *Clostridiales*, *Enterobacteriales*, *Pseudomonadales*, *Bacteroidales*, *Lactobacillales*, *Bacillales*, *Bifidobacteriales*, *Betaproteobacteria*, *Corynebacteriales*, *Micrococcales*, *Selenomonadales*, *Rhizobiales*, *Sphingomonadales*, *Propionibacteriales*, *and Coriobacteriales* abundances were significantly different between HC and BC groups (p < 0.05) (Supplementary Fig. 3D).

At the family level, there were 21 and 16 taxa over 1% in HC and BC, respectively. *Pseudomonadaceae* was dominant in HC with 10.5 ± 8.5%, while *Enterobacteriaceae* and *Ruminococcaceae* were dominant in BC with 23.3 ± 11.2% and 10.8 ± 5.2% abundances, respectively (Fig. 2C). *Enterobacteriaceae* (6.3–23.3%), *Ruminococcaceae* (7.6–10.8%), *Bacteroidaceae* (2.0–9.1%), *Bifidobacteriaceae* (1.3–7.3%), and *Veillonellaceae* (1.0–2.9%) abundances were significantly increased in BC, whereas *Pseudomonadaceae* (10.5–4.8%), *Moraxellaceae* (8.0–3.8%), *Streptococcaceae* (4.5–2.8%), *Lactobacillaceae* (4.3–2.7%), *Staphylococcaceae* (5.2–1.4%), *Burkholderiaceae* (3.7–2.8%), *Corynebacteriaceae* (4.0–1.0%), *Prevotellaceae* (2.3–1.7%), *Micrococcaceae* (2.7–1.2%), and *Sphingomonadaceae* (2.7–0.8%) abundances were significantly decreased (Fig. 2D).

**Figure 3:**
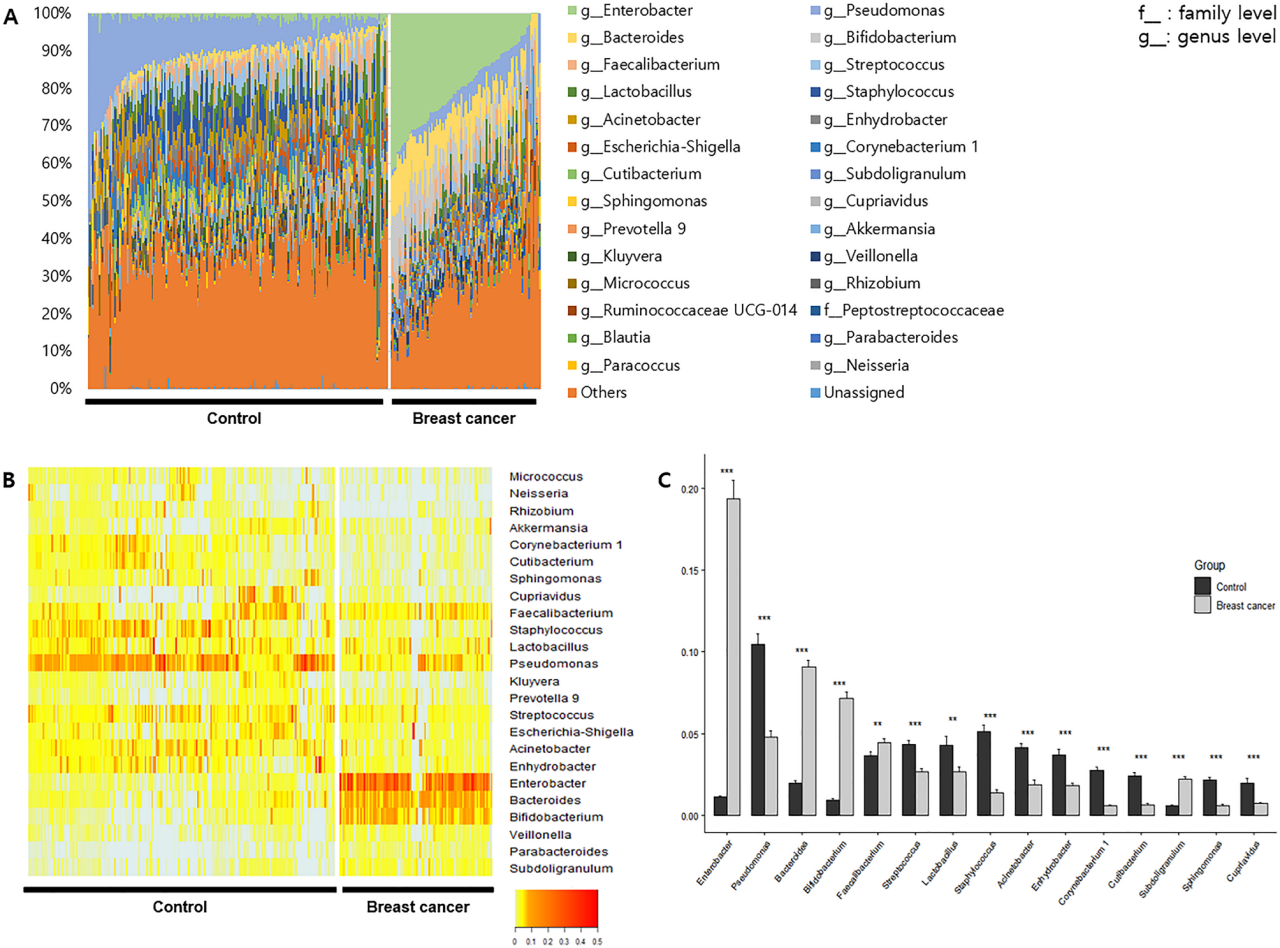
Metagenome profiles in serum of patients with breast cancer and healthy controls at the genus level. (**A**) Abundance of microbiome, (**B**) heatmap of taxa that > 1% of the average abundance in any group, (**C**) significantly different genera.

At the genus level, 20 and 16 genera occupied more than 1% of the abundance in HC and BC, respectively. *Pseudomonas* was dominant in HC (10.5 ± 8.5%), while *Enterobacter* was dominant in BC (19.4 ± 11.1%) (Fig. 3A). *Enterobacter, Bacteroides, Bifidobacterium, Faecalibacterium,* and *Subdoligranulum* abundances were significantly lower in HC than in BC, whereas *Pseudomonas*, *Streptococcus*, *Lactobacillus*, *Staphylococcus*, *Acinetobacter*, *Enhydrobacter*, *Corynebacterium 1*, *Cutibacterium*, *Sphingomonas*, *and Cupriavidus* were significantly higher in HC than in BC (p < 0.05). In particular, the fold changes in *Enterobacter, Bacteroides, Corynebacterium 1*, and *Cutibacterium* were 17.25, 4.66, 0.21, and 0.26, respectively (Fig. 3B,C).

At the assigned taxa to species level, *Enterobacter hormaechei*, *Pseudomonas lurida*, *Pseudomonas psychrophile*,* Kluyvera intermedia*,* Micrococcus* sp. DW-1*, Sphingomonas* sp.* 2F2*, and* Rhizobium* sp.* Root483D2* and *Lactobacillus murinus* levels were significantly different between the HC and BC groups (Supplementary Fig. 3E).

**Figure 4:**
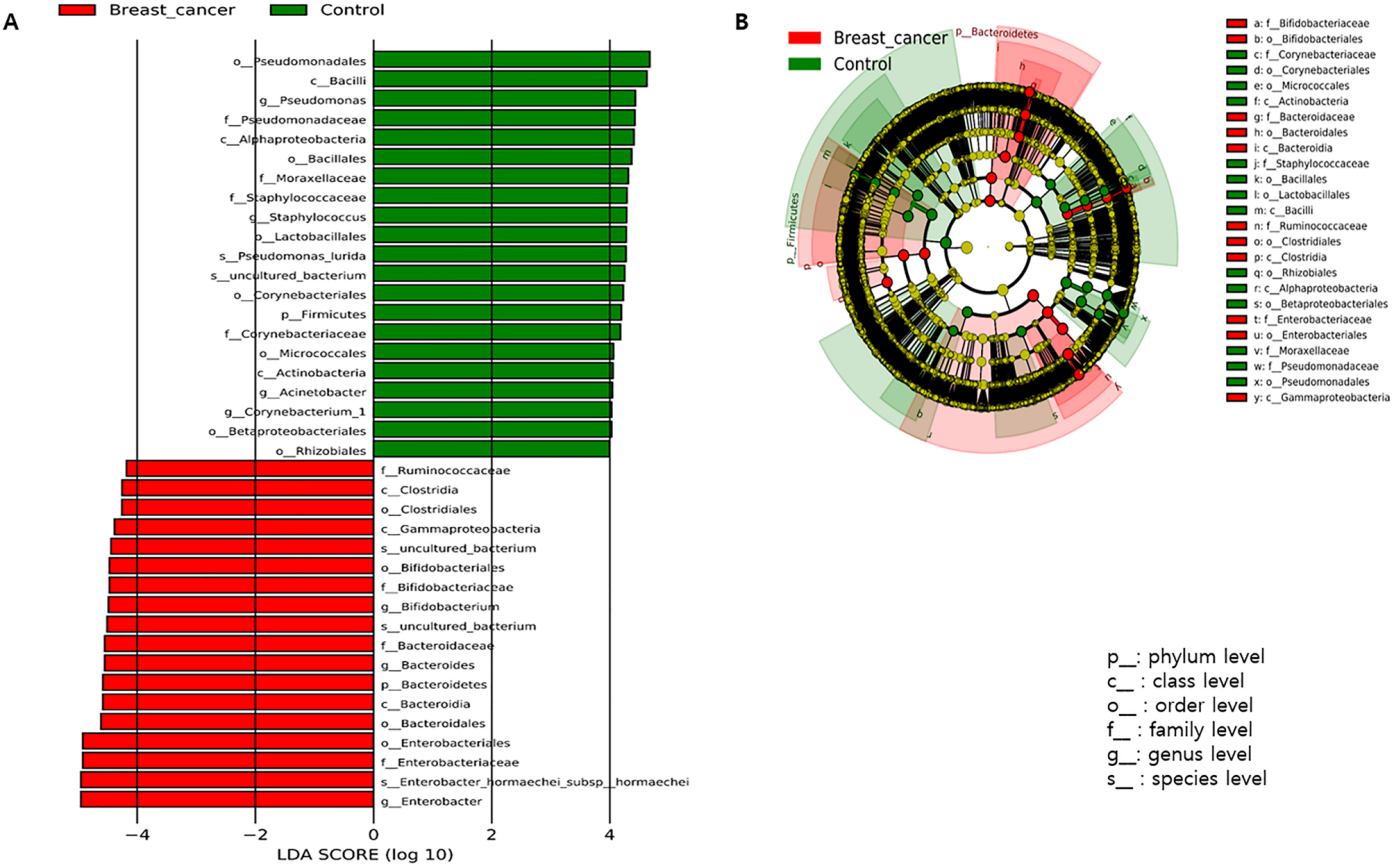
Significantly different taxa between breast cancer and healthy control based on the linear discriminant analysis (LDA) and effect size (LEfSe) analysis. (**A**) LDA score and (**B**) Cladogram.

Significant biomarkers increased in HC based on LEfSe analysis at the genus level included *Pseudomonas, Staphylococcus, Acinetobacter, and Corynebacterium 1*, whereas *Bifidobacterium, Bacteroides,* and *Enterobacter* abundances were decreased in HC. For these genera, log_10_(LDA score) was higher than 4 (Fig. 4A,B).

### Diagnostic models for breast cancer

**Figure 5:**
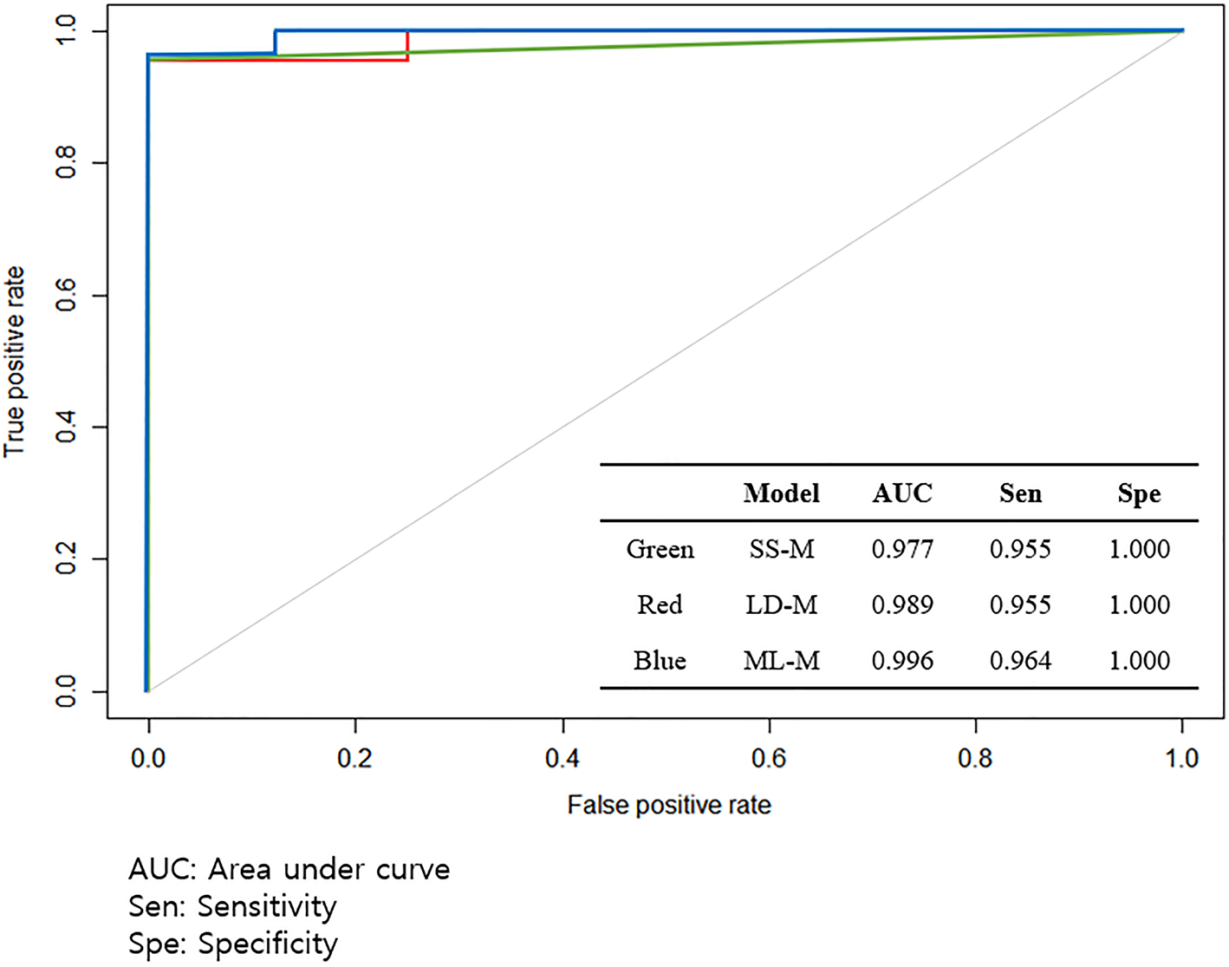
Receiver operating characteristic (ROC) curve of a diagnostic model using test set: SD-M: The model based on significant difference, LD-M: The model based on LEfSe analysis, and ML-M: The model based on machine learning method.

Using EV metagenomic profiles in serum, diagnostic models were developed to distinguish between healthy patients and patients with BC. SD-M and LD-M used logistic regression with stepwise selection for biomarker selection based on significantly different genera and LEfSe analyses, respectively. The SD-M yielded five microbial EV genera as biomarkers: *Enterobacter*, *Bacteroides*, *Kluyvera*, *Pseudomonas*, and *Parabacteroides*. Meanwhile, LD-M revealed six microbial EV genera as biomarkers: *Enterobacter, Pseudomonas*, *Bacteroides*, *Staphylococcus*, *Acinetobacter*, and *Corynebacterium 1*. In the case of ML-M, the relative abundance of total microbial EV metagenomics was input as a variable for analysis rather than specific biomarkers. Model performance was evaluated using test sets based on AUC, sensitivity, and specificity. The resulting BC diagnostic models all revealed AUCs higher than 0.97 and specificity with a value of 1.00. ML-M showed a higher AUC and sensitivity than those of SD-M and LD-M (Fig. 5). In addition, there were no alterations in the AUC, sensitivity, or specificity when age was incorporated as a covariate.

### Relationship between breast cancer and diet

**Figure 6:**
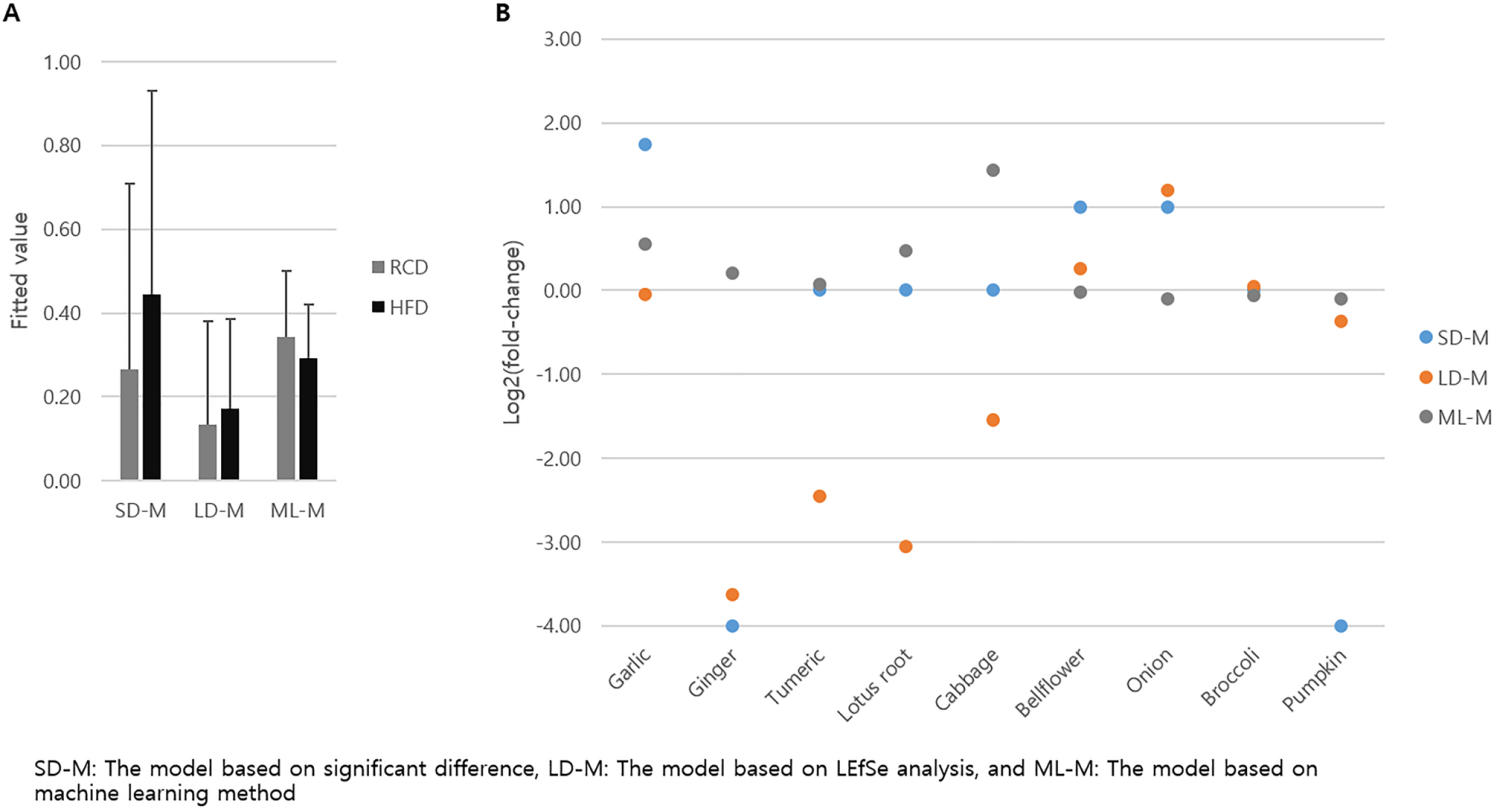
The predicted values of breast cancer risk in mice fed diets using diagnostic models. (**A**) Difference of fitted values between regular chow diet (RCD) and high-fat diet (HFD) and (**B**) Fold-changes between HFD and HFD + diet with garlic, ginger, turmeric, lotus root, cabbage, bellflower root, onion, broccoli, and pumpkin.

To assess the relationship between serum microbial EV-associated BC and diet, the serum EV microbiome of mice treated with RCD, HFD, and HFD + diet was analyzed. Table 1 shows the alterations in the biomarkers associated with BC. *Enterobacter* and *Bacteroides* abundances increased significantly in BC after HFD intervention (p < 0.05). In addition, *Staphylococcus* and *Acinetobacter* abundances decreased in BC after HFD intervention; however, these were not significant. *Enterobacter* abundance was significantly decreased by the addition of ginger, onion, and pumpkin, and that of *Bacteroides* was significantly decreased by the addition of pumpkin, compared to HFD group (p < 0.05). *Bifidobacterium* abundance was decreased by the supplementation of onion and pumpkin by 0.3-fold; however, it was not significant. *Pseudomonas* abundance was significantly increased by the addition of turmeric (p < 0.05), and then increased through pumpkin and ginger by over fourfold, but not significantly. There was no significant alteration in the abundances of *Staphylococcus, Acinetobacter,* and *Corynebacterium 1* between HFD and HFD + diet; however, *Acinetobacter* abundance was drastically increased through onion and pumpkin by fourfold and 2.1-fold, respectively, and that of *Corynebacterium 1* was increased after bellflower root intervention by 3.1-fold. The serum bacterial EV composition differed significantly between the different dietary groups, and the fitted values obtained through each model were also subsequently altered. However, the difference between the dietary effects of RCD and HFD on breast cancer risk was not statistically significant (Fig. 6A). The absolute and negative/positive values of fold-changes in the fitted model values between HFD and HFD + diet groups differed between the models. Breast cancer risk drastically decreased with the addition of ginger in both SD-M and LD-M. Supplementation with pumpkin, turmeric, lotus root, and cabbage reduced the BC risk in HFD-fed mice through SD-M and LD-M, respectively. However, the addition of cabbage increased the risk of BC through ML-M. In addition, the breast cancer risk was increased by the addition of garlic, bellflower root, and onion (Fig. 6B).

**Table 1:** Significant alteration of relative abundance in mouse serum microbiome composition of genera in clinical tests after dietary intervention. Test 1: regular chow diet (RCD) vs. high fat diet (HFD), Test 2: HFD vs. HFD + diet.

Taxon	Test 1		Test 2									
	RCD	HFD	HFD	Garlic	Ginger	Turmeric	Lotus root	Cabbage	Bellflower root	Onion	Broccoli	Pumpkin
*Enterobacter*	0.30 ± 0.32	0.82 ± 0.66	0.31 ± 0.14	0.32 ± 0.42	0.01 ± 0.01	0.51 ± 0.73	0.51 ± 0.45	0.48 ± 0.47	0.06 ± 0.12	0.03 ± 0.05	0.35 ± 0.40	0.00 ± 0.00
*Bacteroides*	0.80 ± 1.06	3.18 ± 2.69	5.94 ± 5.50	7.23 ± 6.42	4.08 ± 3.61	1.91 ± 2.61	2.47 ± 0.70	4.28 ± 3.82	5.84 ± 5.79	5.29 ± 5.82	7.02 ± 2.19	0.82 ± 1.17
*Bifidobacterium*	4.41 ± 2.46	4.55 ± 2.42	4.80 ± 4.25	9.87 ± 13.34	4.90 ± 4.31	7.69 ± 7.16	10.59 ± 7.07	11.38 ± 7.65	3.51 ± 3.70	5.57 ± 4.53	1.62 ± 1.84	1.63 ± 2.99
*Pseudomonas*	2.20 ± 3.22	2.27 ± 1.78	1.44 ± 2.11	1.22 ± 1.57	5.19 ± 4.80	8.84 ± 5.14	2.16 ± 1.38	1.92 ± 1.81	1.03 ± 1.62	1.82 ± 1.13	0.19 ± 0.14	6.15 ± 10.22
*Staphylococcus*	1.23 ± 1.38	0.97 ± 0.79	2.16 ± 1.3	1.76 ± 1.71	2.47 ± 1.70	2.18 ± 2.09	1.46 ± 1.24	1.49 ± 2.18	0.56 ± 1.24	1.17 ± 1.24	0.24 ± 0.20	2.09 ± 1.59
*Acinetobacter*	4.62 ± 8.60	4.20 ± 3.41	6.50 ± 7.19	3.42 ± 2.25	4.32 ± 1.93	5.43 ± 4.08	1.84 ± 1.46	0.83 ± 0.74	4.50 ± 4.93	26.17 ± 31.12	0.25 ± 0.33	13.46 ± 20.37
*Corynebacterium 1*	0.56 ± 0.71	0.97 ± 1.19	1.52 ± 1.79	1.23 ± 0.90	2.13 ± 3.00	0.56 ± 0.76	2.99 ± 4.07	0.78 ± 0.29	4.75 ± 4.69	0.94 ± 1.15	0.35 ± 0.35	1.51 ± 1.25

## Discussion

In this study, we built a predictive model for BC diagnosis using the data of patients with BC and healthy controls recruited from multiple institutions. BC-related microbiome data were used for this model, and a machine learning data analysis was performed. This model considers the presence or absence of BC, age, and specific microbiome levels in the blood as basic parameters. This model showed a satisfactory discriminant function (AUC, 0.99), which was better than that of conventional statistical analysis. In addition, this model can identify microbiomes that are at risk of BC and establish a high-risk group for BC.

Customized local and systemic therapies for BC have improved treatment outcomes and survival rates in patients with BC^18^. Despite these treatments, BC is the most common cancer among women in Korea^2^. These results show the importance of screening and prevention of high-risk groups as well as the treatment of BC. However, conventional tests, such as mammography and ultrasonography, are useful to diagnose cancer after it develops, and these test goals include early screening for breast cancer^19^. The use of a personalized surveillance program prior to disease diagnosis can more accurately predict the risk of BC. Based on this program, we attempted to identify prebiotics that could help prevent BC. Machine learning algorithms can identify the patterns of patients with BC in microbiome data, providing an opportunity to improve the risk prediction of BC compared to traditional methods. The algorithms in this AI-based approach will provide useful analytics for identifying BC risk when used in health-screening centers.

The baseline parameters for this model were age and microbiome type. There was a clear difference in the microbiome abundances between BC and healthy control groups, and *Bacteroidetes* were more abundant at the phylum level of BC in comparison with *Firmicutes*. At the genus level in BC, four types of microbes were dominant, which was reflected in the ROC curve. In addition, the cut-off value was decided to select powerful biomarkers which showed the best performance of the diagnostic models developed using the biomarkers. The ROC curve was used to set the cut-off value and all of the log (LDA score) of the biomarkers used in the diagnostic model with the highest AUC were higher than 4. Therefore, this study showed that 4 is optimal cut-off value for diagnostic models. Previous studies set the cut-off value at 2 or 3 for binary analysis^20,21^. However, in this study, the biomarkers of the best performance model showed that 4 is the optimal cut-off value.

There are many reasons for the differences in the microbiome abundance, but one of them is probably the origin of food. Consumption of different kinds of prebiotics by mice resulted in a shift in the microbiome abundance, even when no external bacteria were added. In other words, the gut microbiome abundance depends on the host's diet, and a specific prebiotic may also create an environment where certain microbes, such as bacteria that prevent BC, predominate. The type of microbiome differs according to the type of food^22^, and the transition to the microbiome of the healthy control group or the BC group was confirmed through diet. *Enterobacter*, *Bacteroides,* and *Bifidobacterium* were more abundant in patients with BC, and *Pseudomonas*, *Staphylococcus*, *Acinetobacter*, and *Corynebacterium 1* were more abundant in healthy controls. Analysis of the microbiome by food group through animal experiments on these bacteria revealed that ginger, onion, and pumpkin lowered microbiome abundance in patients with BC (Table 1). The models developed in this study showed 0–1 value range and meant the risk of breast cancer. The fitted values obtained through SD-L and LD-M increased after consumption of HFD and we suggested that HFD is a risk factor for breast cancer. In addition, the risk of HFD was proved in a previous study^23^. According to the algorithm, we showed that consumption of ginger, pumpkin, turmeric, lotus root, and cabbage decreased fitted value meaning the risk of breast cancer, especially adding ginger to SD-M and LD-M dramatically reduced the risk of breast cancer (Fig. 6B). The previous study also showed that low-fat diet, such as vegetable, fruit, and grain, may reduce the risk of breast cancer^24^.

The microbiome is emerging as a potential target for personalized medicine as it provides exciting solutions for some diseases^25^. Likewise, these three food groups are thought to be capable of inducing positive changes in the microbiome of patients with BC. Bellflower root and broccoli lowered the abundances of Pseudomonas and Staphylococcus which are abundant in healthy controls (Table 1). When the algorithm considered not only information on patients with breast cancer but also that on healthy controls through ML-M, adding cabbage increased the risk of BC the most (Fig. 6B). These results can be used as a reference for future clinical trials.

This study had some limitations. First, study participants in the cohort were enrolled exclusively in Korea. In addition, the incidence rate of BC in Korea is different from that in the West, and its incidence in young women is higher than that in Western countries^26^. Therefore, caution should be exercised when generalizing these results. Second, this algorithm does not take into account genetic problems, which account for 5–10% of BC cases^3^. Third, the pathogenesis of BC has not yet been elucidated, although there are some risk factors. Other environmental problems, such as pregnancy-associated factors, hormonal effect, and lifestyle factors should be considered^27^. In this study, these factors and BMI were excluded from the algorithm due to lack of clinical information of the control group. However, the microbiome itself already reflects these genetic and environmental factors according to previous study^28^. In addition, since there are only animal studies, these results alone cannot be a one-sided conclusion for patients with BC and need to be studied further. We plan to conduct clinical trials in the future so that patients with BC have options to choose more reliable foods.

In summary, this study developed a model to predict BC using a machine-learning approach. Microbiome data were analyzed using a multi-institutional database in Korea, and its usefulness was verified through animal studies. This easily adaptable model can identify high-risk groups and guide individualized surveillance strategies prior to diagnosis using commonly used imaging. Further improvement in this model is expected with additional data, such as genetic and other risk factors. Above all, this study is significant in that it identified the prebiotics that prevent BC.

## Materials and methods

### Patient characteristics

In total, serum samples from 96 patients with BC and 192 healthy controls (HC) were obtained from Ewha Womans University Hospital and Inje University Haeundae Hospital, respectively (Table 2). All participants were Korean females and the mean age of patients with BC was 51.5 years (median 50 years) and that of healthy controls was 51.8 years (median 50 years). Healthy controls were screened using a general health examination. Each patient with BC showed symptoms or abnormal radiologic findings, leading them to visit the hospital for treatment. Among patients with breast cancer recruited, hormone receptor-positive subtypes, luminal A and B, were 68 (70.8%), and hormone receptor-negative subtypes, HER2 and TNBC, were 28 (28.6%). The proportion of patients with hormone-positive breast cancer in all breast cancers is about 70%^29^, which is similar to the proportion of recruited patients at 70.8%. After the patient was histologically diagnosed with breast cancer, blood was collected before undergoing treatments such as surgery, chemotherapy, or radiation therapy. None of the patients included in this study were diagnosed as diabetic or alcoholic, and smokers were not included in this study. Based on the World Health Organization (WHO) criteria of body mass index (BMI), 5 patients (7.2%) were below 18.5 (underweight), 64 patients (66.6%) were within the normal range of 18.5–24.9, 21 patients (21.8%) were 25.0–29.9 (pre-obesity), and 6 patients (6.2%) were Obesity class I of 30.0–34.9. Patients in the normal weight and pre-obesity accounted for 86.6% of the total. This study was approved by the Institutional Review Board of Ewha Womans University Hospital (IRB No. EUMC 2014-10-005) and Inje University Haeundae Hospital (IRB No. 1297992-2015-064). All methods in this study were conducted by the approved guidelines, and informed consent was obtained from all patients. All collected human serum samples were transferred to serum separator tubes (SST) and centrifuged at 3000 rpm for 15 min at 4 °C.

**Table 2:** Patient characteristics.

	Healthy control	Breast cancer
Female (total N)	192	96
Age (year)	51.8 ± 10.1	51.5 ± 11.1
Pathologic feature, N (%)		
Tumor size		
Tis		3 (3.1)
T1		54 (56.2)
T2		35 (36.4)
T3		4 (4.1)
Clinical node status		
N0		64 (66.6)
N1		22 (22.9)
N2		7 (7.2)
N3		3 (3.1)
Tumor grade		
G1		1 (1.0)
G2		50 (52.0)
G3		45 (46.8)
Ki67		
˂ 20%		45 (46.8)
≥ 20%		51 (56.2)
Clinical stage		
0		3 (3.1)
I		44 (45.8)
II		36 (37.5)
III		13 (13.5)

### In vivo mouse study model

Six-week-old female C57BL/6 mice at 6 weeks of age (Orient Bio Inc., Seongnam, Korea) were used in this study. The mice were housed and maintained under standard laboratory conditions at 22 ± 2 °C and 50 ± 5% humidity under 12 h day and night cycles throughout the course of the in vivo study. The animal study was approved by the Institutional Animal Care and Use Committee of Chung-Ang University (Approval No. 2018-00057). All methods in this animal study were conducted in accordance with the approved guidelines.

### Evaluation of dietary effects

To analyze dietary effects, in vivo testing was conducted twice: before and after dietary intervention. First, the mice were randomly divided into two groups (n = 60): an RCD group fed a regular chow diet (RCD) and an HFD group fed a high-fat diet (HFD). Second, the mice were randomly divided into 11 groups (n = 5) due to prebiotics types including an RCD group, HFD group, and HFD + diet group fed HFD supplemented with garlic (*Allium sativum*), ginger (*Zingiber officinale*), turmeric (*Curcuma longa*), lotus (*Nelumbo nucifera*) root, cabbage (*Brassica oleracea var. capitata*), bellflower (*Platycodon grandiflorum*) root, onion (*Allium cepa*), broccoli (*Brassica oleracea var. italica*), and pumpkin (*Cucurbita moschata*). The mice in RCD control group were fed regular chow containing 18% dietary fat (Research Diets, Inc., New Brunswick, NJ, USA) for four weeks. The mice in HFD group were fed a 60% fat diet (Research Diets, Inc.), and orally administered diet powder (100 µg) once daily for 4 weeks. At the conclusion of the 4-week study period, all mice were sacrificed and serum was collected under ketamine/xylazine anesthesia.

### EV DNA extraction and sequencing

To extract EVs from serum, centrifugation, filtering, and boiling were performed as previously described^15^. Serum EV DNA was extracted using the DNeasy PowerSoil kit (QIAGEN, Germany). Finally, the extracted EV DNA from each sample was quantified using the QIAxpert (QIAGEN, Germany). Isolated EV microbial genomic DNA was amplified by targeting 16S V3-V4 hypervariable regions as primers: 16s_V3_F(5′-TCGTCGGCAGCGTCAGATGTGTATAAGAGACAGCCTACGGGNGGCWGCAG-3′) and 16s_V4_R (5′-GTCTCGTGGGCTCGGAGATGTGTATAAGAGACAGGACTACHVGGGTATCTAATCC-3′)^16,17^. Libraries were prepared using PCR products, and all amplicons were sequenced using MiSeq (Illumina, USA).

### Metagenomic analysis of microbial EV composition

Taxonomic assignments were performed using the profiling program MDx-Pro ver.2 (MD Healthcare, Korea). Briefly, paired-end reads were filtered according to the barcode, and primer sequences were trimmed using Cutadapt (version 1.1.6) and then merged with CASPER. To obtain high-quality sequencing reads, sequences with read lengths less than 350 bp or over 550 bp and Phred quality scores below 20 were discarded. The VSEARCH de novo clustering method was used to assign Operational taxonomic units (OTUs) to the genus level with a 97% similarity threshold. OTUs containing one sequence in only one sample were excluded from further analyses. Subsequently, taxonomic assignments were conducted at the species level using UCLUST and QIIME 1.9.1 against the Silva 132 database under default parameters. If clusters could not be assigned at the genus level owing to insufficient taxonomic information in the database, the taxon was assigned to the next highest level. Brackets around the taxon name represent an unverified suggested a taxonomic assignment based primarily on whole-genome phylogeny within the genomic database.

### Predictive diagnostic model development

To develop a BC diagnostic model, we considered the relative abundances of OTUs at the genus level as model variables. First, we selected candidate biomarkers with p-values < 0.01, fold-changes greater than twofold, and average relative abundances greater than 1%. The biomarkers included as model variables were selected using methods to determine the model with the highest area under the curve (AUC) value, sensitivity, specificity, and accuracy. The first model (SD-M) used biomarkers with differing variables based on significantly different genera. The biomarkers of the second model (LD-M) were based on linear discriminant analysis (LDA) and LDA effect size (LEfSe). LEfSe was used to select significant biomarkers, and the cut-off of the log (LDA score) was set at 4 by ROC curve. Diagnostic models were calculated using logistic regression with stepwise selection, in which the Akaike information criterion (AIC). We also analyzed age as a covariate, in addition to SD-M and LD-M. The third model (ML-M) was developed using a machine learning algorithm based on the gradient boosting machine (GBM) ensemble method using microbiome composition. GBM was incorporated in the model using the gradient boosting regressor of scikit-learn (version 0.21.3) in Python (version 3.6.9). The diagnostic models were developed with the training and test sets used at an 80:20 ratio for model validation.

### Statistical analysis

Significant differences in the age and microbiome composition in the serum were determined using Student’s t-test or Wilcoxon rank-sum test. Principal coordinate analysis (PCoA) based on the Bray–Curtis dissimilarity distance was conducted to determine individual taxa-level clustering of groups. Permutational multivariate analysis of variance (PERMANOVA) was used to analyze the p-values for the PCoA. LEfSe was also used to select significant biomarkers, and the cut-off of the log (LDA score) was set at 4. The results were considered significant when p-values were less than 0.05 (p < 0.05) and all analyses were conducted using R version 3.6.1.

## Supplementary Information

Supplementary Figures.

## Data Availability

The raw sequence data and processed data of metagenome analysis are available through the Sequence Read Archive under BioProject ID: PRJNA834579, PRJNA834581, and PRJNA834582.
