# Supplementary material for: Prediction of breast cancer using blood microbiome and identification of foods for breast cancer prevention
Source: Sci Rep. 2023 Mar 29;13:5110. doi: 10.1038/s41598-023-32227-x (PMC10060235; doi:10.1038/s41598-023-32227-x)
Supplement: Supplementary file 1 — Supplementary Figures. [file 41598_2023_32227_MOESM1_ESM.pdf]

Supplementary Fig 1. Rarefaction curve based on Chao1 index

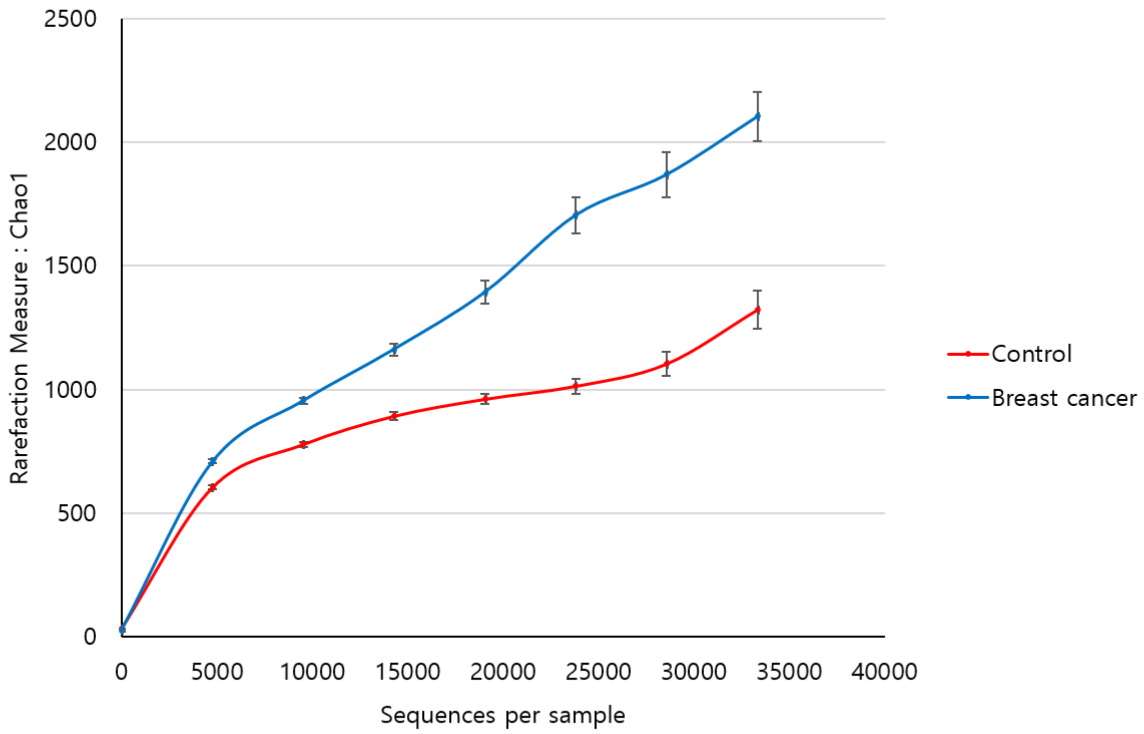

Supplementary Fig 2. Beta diversity based on Principal Coordinate Analysis (PCoA) at the A) class level, B) Order level, and C) Species level

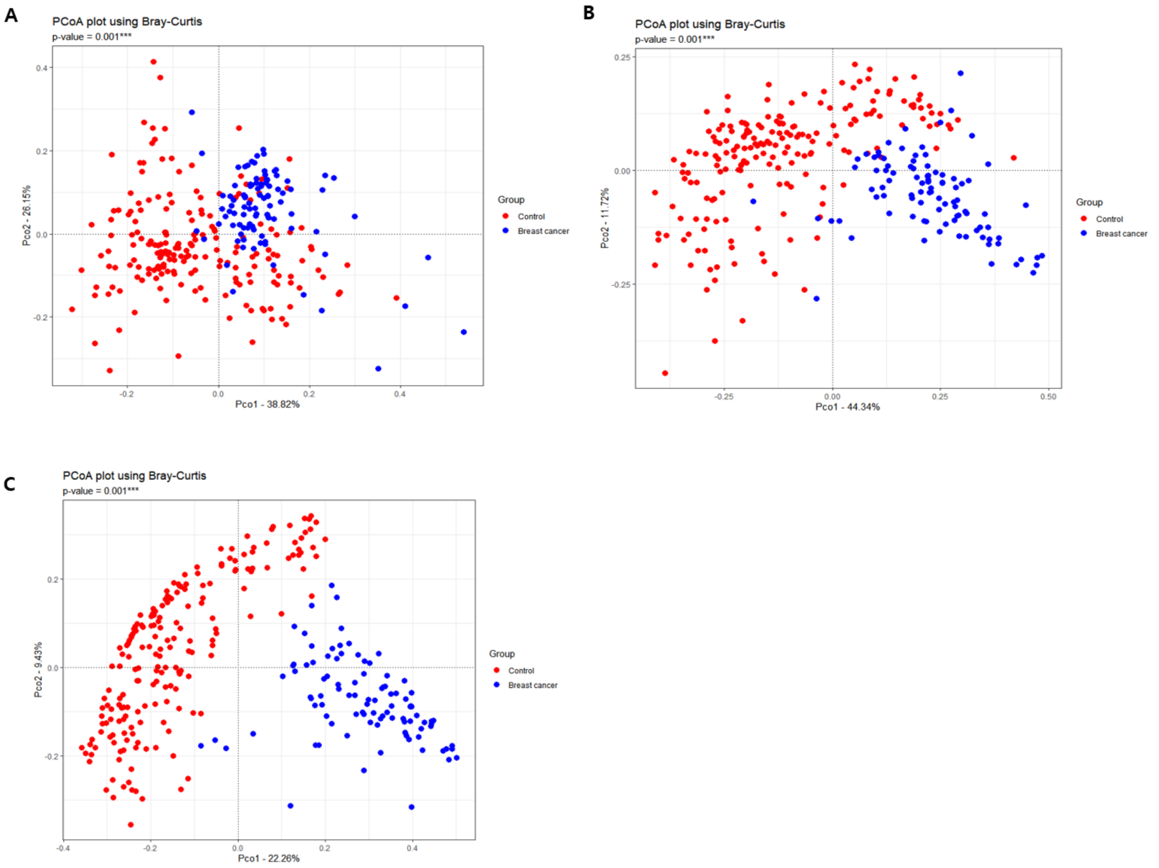

Supplementary Fig 3. Difference of microbiome in serum between breast cancer patients and healthy controls. A) Composition of microbiome at the class level, b) Significantly different taxa at the class level, C) Composition of microbiome at the order level, D) Significantly different taxa at the order level, and E) Significantly different taxa at the species level

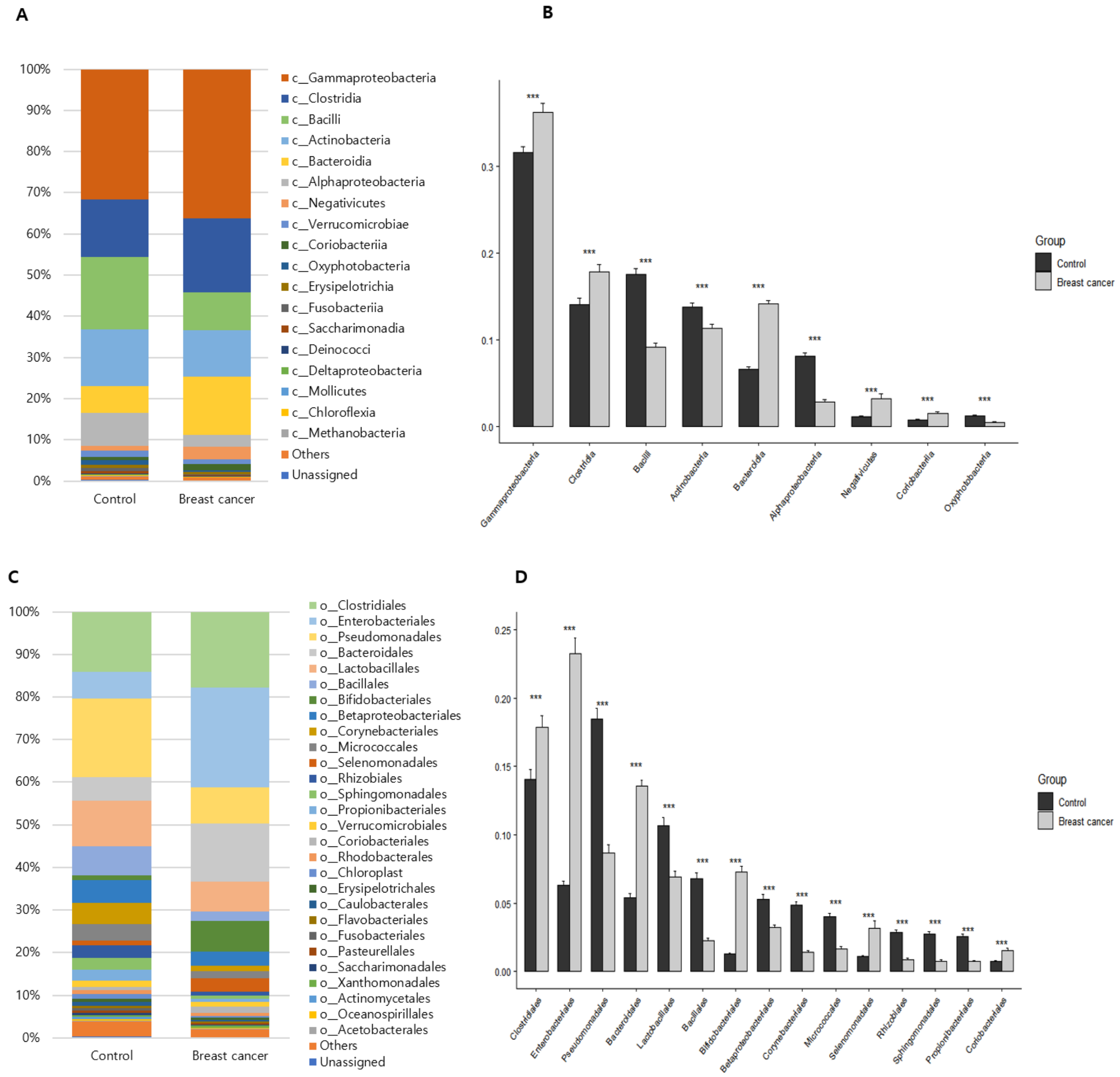

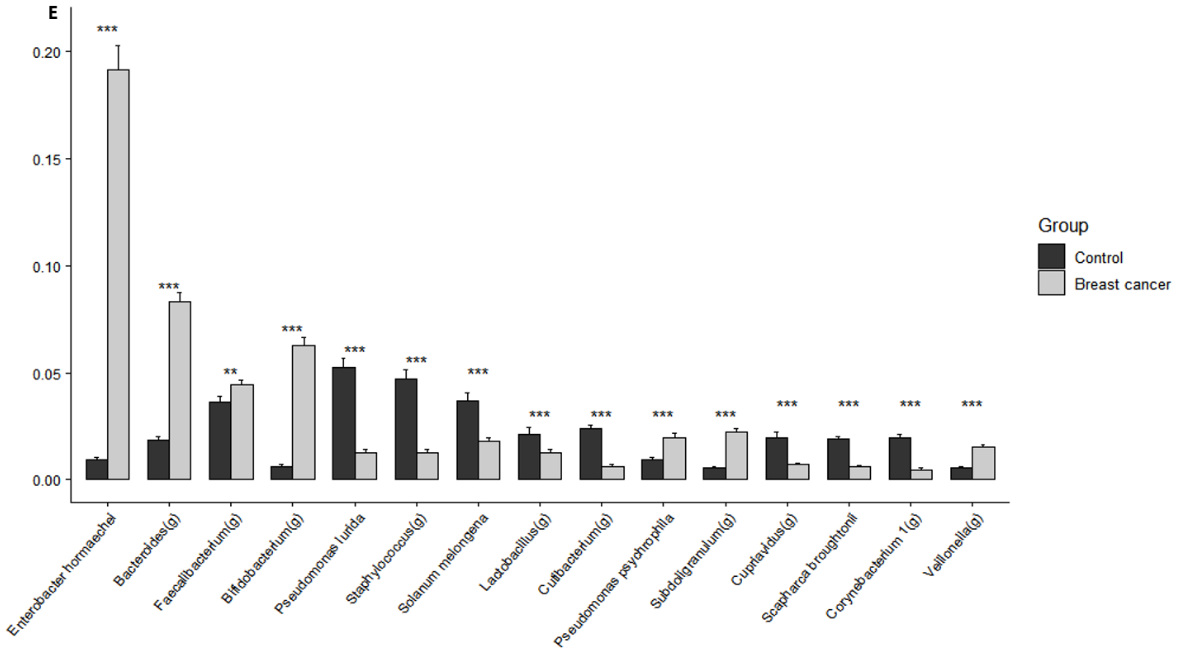
E
0.20
0.15
0.10
0.05
0.00
Enterobacter hormaechei
Bacteroides(g)
Faecalibacterium(g)
Bifidobacterium(g)
Pseudomonas lurida
Staphylococcus(g)
Solanum melongena
Lactobacillus(g)
Cutibacterium(g)
Pseudomonas psychrophila
Subdoligranulum(g)
Cupriavidus(g)
Scapharca broughtonii
Corynebacterium 1(g)
Veillonella(g)
Group
Control
Breast cancer
